# Supplementary material for: Serving Temperatures of Best-Selling Coffees in Two Segments of the Brazilian Food Service Industry Are “Very Hot”
Source: Foods. 2020 Aug 3;9(8):1047. doi: 10.3390/foods9081047 (PMC7466196; doi:10.3390/foods9081047)
Supplement: Supplementary file 1 [file foods-09-01047-s001.pdf]

SUPLEMENTARY MATERIAL

Table S1 Estimates of per capita (15+) daily intakes of coffee (in ml of beverage) in Brazil compared to selected countries and world regions, 2015 and 2019.

Country or World Region	Population (15+) [1]		Consumption of green coffee by country or world region in kg [3]		Consumption of green coffee in kg/person/year [4]		Consumption of roasted coffee in kg/person/year [5]		Annual intake of coffee in ml/person [6]		Daily intake of coffee in ml/ person [7]	
	2015	2019 [2]	2015	2019	2015	2019	2015	2019	2015	2019	2015	2019
Brazil	158,627,509	168,540,057	1,230,480,000	1,325,340,000	7.76	7.86	6.52	6.61	81,482	82,601	223	226
World	5,448,471,219	5,811,150,103	9,329,460,000	9,869,220,000	1.71	1.70	1.44	1.43	17,986	17,840	49	49
Europe	625,629,041	627,565,932	3,128,820,000	3,336,900,000	5.00	5.32	4.20	4.47	52,532	55,853	144	153
Africa	695,955,074	799,767,716	657,060,000	588,000,000	0.94	0.74	0.79	0.62	9,917	7,723	27	21
Asia & Oceania	3,373,371,984	3,584,022,598	1,971,780,000	2,116,560,000	0.58	0.59	0.49	0.50	6,140	6,203	17	17
Central America and Mexico	120,275,359	131,357,633	317,700,000	317,640,000	2.64	2.42	2.22	2.03	27,746	25,401	76	70
North America	289,562,407	302,083,790	1,736,040,000	1,894,680,000	6.00	6.27	5.04	5.27	62,977	65,883	173	181
South America	311,754,735	333,241,715	1,517,940,000	1,615,440,000	4.87	4.85	4.09	4.07	51,145	50,921	140	140
USA	259,193,542	270,191,676	1,520,160,000	1,663,920,000	5.86	6.16	4.93	5.18	61,607	64,688	169	177
Argentina	32,215,601	34,152,047	32,760,000	34,440,000	1.02	1.01	0.85	0.85	10,682	10,593	29	29
Colombia	35,864,007	39,595,244	104,160,000	106,500,000	2.90	2.69	2.44	2.26	30,507	28,253	84	77
Japan	111,360,657	110,731,963	467,400,000	451,380,000	4.20	4.08	3.53	3.43	44,088	42,819	121	117
Switzerland	7,360,004	7,069,406	64,140,000	64,440,000	8.71	9.12	7.32	7.66	91,541	95,749	251	262
Norway	4,265,817	4,485,693	46,440,000	54,240,000	10.89	12.09	9.15	10.16	114,354	127,014	313	348

[1] Populations aged 15 years and older (15+) for selected countries and world regions were obtained from the Population Division (by age group) of the United Nations [8]. [2] Once the populations by age groups from the UN are available at 5 years intervals only (e.g. 2005, 2010, 2015 and 2020), the 15+ populations in 2019 corresponds to UN estimates of the 15+ population in 2020. [3] Coffee consumption data by country or world region was obtained from the International Coffee Organization (ICO) [7] and it was assumed all ICO weight data is in green coffee form. [4] Consumption of green coffee (in kg/person/year) was obtained by dividing the consumption of green coffee in kg by the population (15+) of country or world region. [5] Consumption of roasted coffee (in kg/person/year) was obtained by applying a 1.19 conversion factor to roasted coffee (1.19 kg green coffee = 1.0 kg of roasted coffee), according to ICO [9]. [6] Annual intake of coffee (in ml/person) was obtained by applying a coffee-to-water ratio of 1 to 12.5 (8 g roasted coffee/100 ml water) [10, 11] to the consumption of roasted coffee (in kg/person/year) x 1,000. [7] Daily intakes of coffee (in ml/person) were obtained by dividing the annual intake of coffee (in ml/person) by 365.
